# Cost-effectiveness analysis of first-line sintilimab plus chemotherapy vs. chemotherapy alone for unresectable advanced or metastatic gastric or gastroesophageal junction cancer in China

**DOI:** 10.3389/fphar.2024.1411571

**Published:** 2024-09-04

**Authors:** Zuojuan Xiang, Ling Ma, Yingzhou Fu, Yong Pan

**Affiliations:** ^1^ Department of Pharmacy, the Affiliated Cancer Hospital of Xiangya School of Medicine, Central South University, Hunan Cancer Hospital, Changsha, China; ^2^ Department of Clinical pharmacy, the First People’s Hospital of Yunnan Province, the Affiliated Hospital of Kunming University of Science and Technology, Kunming, China

**Keywords:** cost-effectiveness, sintilimab, gastric cancer, gastroesophageal junction cancer, partitioned survival approach

## Abstract

**Background:**

The Chinese Society of Clinical Oncology (CSCO) has recommended sintilimab plus chemotherapy (SINT + Chemo) as a standard first-line therapy for advanced gastric cancer or gastroesophageal junction cancer (GC/GEJC), based on the proven effectiveness and safety in the ORINT-16 trail. Its cost-effectiveness, however, remains to be evaluated.

**Methods:**

We established a partitioned survival approach (PartSA) model with a 10-year time horizon to determine whether SINT + Chemo (vs. chemotherapy) was more cost-effective as a first-line treatment for unresectable advanced or metastatic GC/GEJC. Survival data was generated from the ORIENT-16 trail. Cost calculation was limited to direct medical costs. Database of Hunan Public Resources Trading Service Platform was used as the source for obtaining drug prices. Other cost and utility values were gathered from established literature. Incremental cost-effectiveness ratio (ICER) was the primary output. Additionally, we conducted sensitivity analysis, subgroup analysis, and scenario analysis.

**Results:**

In the base-case analysis, group SINT + Chemo showed an increase in utility value by 0.32 quality-adjusted life-years (QALYs) at an extra cost of $7988.43, resulting in an ICER of $25239.29/QALY, below the Chinese cost-effective willingness-to-pay (WTP) threshold of $38223.34. Upon further subgroup analysis according to patients’ programmed death 1 ligand (PD-L1) combined positive score (CPS), the ICERs were $26341.01/QALY for patients highly expressing PD-L1 (CPS ≥5) and $17658.26/QALY for patients lowly expressing PD-L1 (CPS <5). Based on the sensitivity analysis, we found the PFS utility was the parameter that had the most significant impact on the model’s outcomes. Moreover, in scenario analysis, the results remained consistent despite variations in the model’s time frame.

**Conclusion:**

In China, SINT + Chemo is a more cost-effective option (vs. chemotherapy) as a first-line therapy for unresectable advanced or metastatic GC/GEJC, irrespective of PD-L1 expression levels.

## 1 Introduction

World-wide, gastric cancer or gastroesophageal junction cancer (GC/GEJC) ranks fourth in cancer-related mortality and fifth in malignant neoplasm prevalence, while around 44% of GC/GEJC cases are found in China (Sung et al., 2021). In 2022, 358,700 new cases were identified, and 260,400 patients died from it, reported by the National Cancer Center of China (Zheng et al., 2024). Up to 80% of patients with GC/GEJC have progressed to the advanced stage when diagnosed, with less than 1 year’s median survival (Büyükkaramikli et al., 2017; Wagner et al., 2017; Smyth et al., 2020). In spite of a declining trend in incidence and mortality, the burden of GC/GEJC remains significant (Smyth et al., 2020).

Before molecular therapeutics were introduced, fluoropyrimidine and platinum based chemotherapy for advanced GC/GEJC were found to have limited effectiveness. Currently, the preferred treatment for advanced ERBB2 (formerly HER2)-positive GC/GEJC involves ERBB2-targeted agents in combination with chemotherapy, which has demonstrated significant survival benefits. However, as more than 80% of GC/GEJC are ERBB2-negative (Van Cutsem et al., 2016), a new approach is still needed. Recently, immune checkpoint inhibitor (ICI) plus chemotherapy has shown promising results in treating patients with advanced ERBB2-negative GC/GEJC, making it a new first-line treatment for this subset of patients (Shitara et al., 2020; Janjigian et al., 2021; Akkanapally et al., 2024). One of these is sintilimab, a fully humanized monoclonal antibody against the programmed cell death-1 (PD-1). Due to the positive outcomes seen in multiple clinical trials, sintilimab has been authorized for treating several forms of cancer in China (Shi et al., 2019; Ren et al., 2021; Zhou et al., 2021; Lu et al., 2022; Xu et al., 2023). The ORIENT-16 study (NCT03745170) was a phase III clinical trial that was randomized and double-blind, enrolling patients with GC/GEJC from 62 hospitals in China (Xu et al., 2023). The findings indicated that sintilimab plus chemotherapy (SINT + Chemo) brought significant prolongations in both overall survival (OS) and progression-free survival (PFS). In the overall population and patients highly expressing programmed death 1 ligand (PD-L1) with combined positive score (CPS) ≥ 5, the median OS was 15.2 vs. 12.3 months (hazard ratio (HR) = 0.77, 95% confidence interval (CI): 0.63–0.94) and 18.4 vs. 12.9 months (HR = 0.66, 95% CI: 0.50–0.86), respectively. The corresponding median PFS was 7.1 vs. 5.7 months (HR = 0.64, 95% CI: 0.52–0.77) and 7.7 vs. 5.8 months (HR = 0.63, 95% CI: 0.49–0.81). Among patients lowly expressing PD-L1 with CPS <5, the median PFS was 7.0 vs. 5.6 months (HR = 0.66, 95% CI: 0.49–0.89) according to the *post hoc* analysis. Despite this, no substantial enhancement in OS was observed, accordant with the findings of the CheckMate 649 trial, which evaluated nivolumab’s efficacy (Janjigian et al., 2021). As a result, since 2022, the Chinese Society of Clinical Oncology (CSCO) has recommended SINT + Chemo as a standard first-line treatment for advanced GC/GEJC.

Although ICI plus chemotherapy has established clinical effectiveness and safety as the regimen for GC/GEJC, it is crucial to assess its evidence of cost-effectiveness, as the higher costs associated with combination therapy may pose a significant economic burden to the healthcare system. However, current pharmacoeconomic evaluations mainly focus on nivolumab, pembrolizumab and tislelizumab (Jiang et al., 2022; Shu et al., 2022; Cao et al., 2023; Lang et al., 2023; Morimoto et al., 2023; Zhang et al., 2023; Li et al., 2024), leaving the cost-effectiveness of sintilimab unexplored. In this study, the cost-effectiveness of SINT + Chemo vs. chemotherapy as a first-line treatment for GC/GEJC was evaluated from a Chinese healthcare system’s perspective.

## 2 Methods

The research was carried out from a Chinese healthcare system’s perspective, and conformed to CHEERS (Consolidated Health Economic Evaluation Reporting Standards) as outlined in Supplementary Table S1 (Husereau et al., 2022).

### 2.1 Population and intervention

The patient characteristics and interventions employed in this model were based on the ORIENT-16 trial (Xu et al., 2023). Since there were no human subjects directly participating, there was no need for a review by an institutional review board or an exemption from an ethics committee during this study. Eligible patients needed to be aged 18 or older and confirmed unresectable locally advanced or metastatic GC/GEJC, along with at least one measurable or evaluable lesion according to Response Evaluation Criteria in Solid Tumors (RECIST) version 1.1, performance status of 0 or 1 according to the Eastern Cooperative Oncology Group (ECOG), and adequate hematologic, hepatic and renal function. Patients previously treated with radiotherapy or adjuvant/neoadjuvant chemotherapy were allowed if their disease reoccurred at least 6 months after their last treatment. The main exclusion criteria were previous systemic therapy, confirmed ERBB2-positive status, and presence of autoimmune disease. Patients were categorized into two subgroups based on their PD-L1 CPS values: the high expression group (CPS ≥5) and the low expression group (CPS <5).

An equivalent number of patients were assigned at random to either receive sintilimab or a placebo. Next, either sintilimab (3 mg/kg for those with body weight less than 60 kg or 200 mg for those with 60 kg or more intravenously) or a placebo was given in addition to the XELOX regimen (capecitabine 1,000 mg/m^2^ taken orally twice daily on days 1–14 and oxaliplatin 130 mg/m^2^ given intravenously on day 1) for a total of six cycles lasting 3 weeks each. Afterward, patients were given maintenance treatments with either sintilimab or a placebo along with capecitabine at the same dose.

### 2.2 Model structure

**FIGURE 1:**
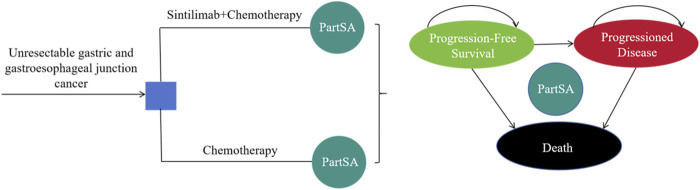
Model structure. PartSA, partitioned survival approach.

We evaluated the cost and effectiveness of treatments for advanced GC/GEJC with a partitioned survival approach (PartSA) model, which were established by using TreeAge Pro 2022. Given the poor prognosis of advanced metastatic stomach cancer, with a mere 5% 5-year survival rate (Büyükkaramikli et al., 2017), the time horizon for this model was established at 10 years. While in order to align with ORIENT-16 trial’s chemotherapy protocol, a 3 weeks cycle length was implemented. This model simulated three health states that were mutually exclusive: PFS, progressive disease (PD), and death. All individuals were assumed to begin in the PFS state and could move to either PD or death status (Figure 1). Incremental cost-effectiveness ratio (ICER) was the primary output, and the cost-effectiveness of the treatment can be judged by comparing it to the specified willingness-to-pay (WTP) value. Following the 2020 version of the China Guidelines for Pharmacoeconomic Evaluations (CGPE) and the World Health Organization’s recommendation (Marseille et al., 2015), treatments should be considered cost-effective if the ICER is between one and three times the gross domestic product (GDP) *per capita* of China and highly cost-effective if it is less than one times. So the threshold for WTP in this study was established at $38,223.34, equivalent to three times the GDP *per capita* of China in 2022.

WebPlotDigitizer was used to extract time-to-survival data from survival curves. Subsequently, R language software (version 4.3.2) was employed to reconstruct individual time-to-event data and extrapolate survival curves based on Guyot et al.‘s algorithm (Guyot et al., 2012). Various parametric survival models, including exponential, weibull, log-normal, log-logistic, gompertz, and generalized gamma, were utilized for curve fitting and extrapolation. The parametric models that had the smallest Akaike Information Criterion (AIC) and Bayesian Information Criterion (BIC) values were determined to be the best-fitted models (Supplementary Table S2; Supplementary Figures S1–S3). Distributions and key parameters of the optimal survival curves are summarized in Table 1.

**TABLE 1:** Parameters of the best-fitted distributions.

Kaplan meier survival curve	Best fitted distribution	Key parameters
Overall population		
OS curve of SINT + Chemo arm	log-logistic	shape = 1.638, scale = 15.666
PFS curve of SINT + Chemo arm	log-logistic	shape = 1.601, scale = 8.559
OS curve of Chemo arm	log-logistic	shape = 1.818, scale = 12.242
PFS curve of Chemo arm	log-logistic	shape = 2.005, scale = 6.065
Patients with PD-L1 CPS≥5		
OS curve of SINT + Chemo arm	log-normal	meanlog = 2.972, sdlog = 1.118
PFS curve of SINT + Chemo arm	log-logistic	shape = 1.722, scale = 9.027
OS curve of Chemo arm	log-normal	meanlog = 2.552, sdlog = 1.033
PFS curve of Chemo arm	log-logistic	shape = 2.204, scale = 6.362
Patients with PD-L1 CPS<5		
OS curve of SINT + Chemo arm	log-logistic	shape = 1.794, scale = 12.239
PFS curve of SINT + Chemo arm	log-logistic	shape = 1.520, scale = 7.776
OS curve of Chemo arm	log-logistic	shape = 2.137, scale = 11.547
PFS curve of Chemo arm	log-normal	meanlog = 1.730, sdlog = 0.840

Chemo, chemotherapy; SINT, sintiliamb.

### 2.3 Inputs of cost and utility

Key inputs of cost and utility are shown in Table 2. Our analysis was limited to direct medical costs, including drug costs, intravenous administration, regular check-ups, imaging procedures, end-of-life care, and expenses related to severe adverse events. After consulting with clinical experts, it was found that there is little disparity in the monitoring plans for these two treatment regimens. Moreover, genetic testing is infrequently carried out before the clinical administration of sintilimab. Therefore, in this study, the costs for routine check-ups and imaging procedures were assumed to be the same for both regimens on a per-cycle basis. Drug prices were sourced from the database of the Hunan Public Resources Trading Service Platform (https://yycg.hnsggzy.com/), reflecting the typical pricing at most public hospitals in China. Drug dosage was determined by using the average body weight of 65 kg and a body surface area (BSA) of 1.72 m^2^ (Qiao et al., 2021). At the same time, dose wastage of sintilimab was considered given that its only specification was 10 mL:100 mg. We sourced other costs from previously published investigations (Qiao et al., 2021; Chen et al., 2022; Shu et al., 2022; Liu et al., 2023). Patients were assumed to undergo second-line treatment once their disease progressed, with details of chemotherapeutic agent proportion and usage available in Supplementary Table S3, including capecitabine, oxaliplatin, paclitaxel, apatinib, pembrolizumab, and nivolumab, based on subsequent anticancer therapy data from the ORIENT-16 trial and CSCO guideline. As adverse events of grade 1–2 can be managed effectively, this study only took into account the adverse events of grade 3 or higher with an incidence higher than 5%. Moreover, adverse events were assumed to occur in the initial cycle. All costs were adjusted to reflect 2022 values using the Consumer Price Index and then converted to American dollars at a rate of 1 USD to 6.7261 CNY. Utility values of PFS/PD state and disutility values due to serious adverse events were acquired from existing literature sources (Chen et al., 2022; Shu et al., 2022). Furthermore, as per the 2020 version of the CGPE, both costs and quality-adjusted life-years (QALYs) were discounted at an annual rate of 5%, and half-cycle correction was applied for the outcomes.

**TABLE 2:** Key model inputs.

Parameter	Base-case value (range)	Distribution	References
Cost ($)			
Routine follow-up cost per cycle	57.06 (45.65–68.48)	Gamma	Shu et al. (2022)
Tests and radiological examination per cycle	99.89 (79.92–119.87)	Gamma	Shu et al. (2022)
Supportive care per cycle	116.35 (93.08–139.62)	Gamma	Shu et al. (2022)
Terminal care	1032.46 (825.97–1238.95)	Gamma	Shu et al. (2022)
Intravenous drug administration per unit	2.79 (2.23–3.35)	Gamma	Shen et al. (2022)
Cost of drugs			
Sintilimab/100 mg	160.57 (128.46–192.68)	Gamma	
Capecitabine/1000 mg	0.9 (0.63–6.55)	Gamma	
Oxaliplatin/100 mg	50.55 (10.94–523.33)	Gamma	
Paclitaxel/100 mg	36.57 (18.83–242.5)	Gamma	
Apatinib/1000 mg	59.43 (47.54–71.32)	Gamma	
Pembrolizumab/100 mg	2663.95 (2131.16–3196.74)	Gamma	
Nivolumab/100 mg	1540.08 (1232.06–1848.1)	Gamma	
Cost of serious adverse events			
Platelet count decreased	1505.92 (1240.17–1771.67)	Gamma	Chen et al. (2022)
Neutrophil count decreased	115.01 (51.11–357.80)	Gamma	Chen et al. (2022)
White blood cell count decreased	467.86 (350.90–584.83)	Gamma	Liu et al. (2023)
Anemia	468.19 (374.5–561.79)	Gamma	Chen et al. (2022)
Utility			
PFS	0.797 (0.64–0.96)	Beta	Shu et al. (2022)
PD	0.577 (0.46–0.69)	Beta	Shu et al. (2022)
Platelet count decreased	0.65 (0.52–0.78)	Beta	Chen et al. (2022)
Neutrophil count decreased	0.2 (0.15–0.5)	Beta	Chen et al. (2022)
White blood cell count decreased	0.2 (0.16–0.24)	Beta	Chen et al. (2022)
Anemia	0.07 (0.05–0.08)	Beta	Chen et al. (2022)
Risk of serious adverse events in SINT + Chemo group (%)			
Platelet count decreased	24.7 (19.76–29.64)	Beta	Xu et al. (2023)
Neutrophil count decreased	20.1 (16.08–24.12)	Beta	Xu et al. (2023)
White blood cell count decreased	7.6 (6.08–9.12)	Beta	Xu et al. (2023)
Anemia	12.5 (10–15)	Beta	Xu et al. (2023)
Risk of serious adverse events in Chemo group (%)			
Platelet count decreased	21.3 (17.04–25.56)	Beta	Xu et al. (2023)
Neutrophil count decreased	18.8 (15.04–22.56)	Beta	Xu et al. (2023)
White blood cell count decreased	6.9 (5.52–8.28)	Beta	Xu et al. (2023)
Anemia	8.8 (7.04–10.56)	Beta	Xu et al. (2023)
Body surface area (m^2^)	1.72 (1.39–2.06)	Gamma	Qiao et al. (2021)
Body weight (kg)	65 (52–78)	Gamma	Qiao et al. (2021)
Discount rate (%)	5 (0–8)	Fix	

Chemo, chemotherapy; SINT, sintiliamb.

### 2.4 Sensitivity analysis

Model robustness was assessed through one-way and probabilistic sensitivity analyses. The former examined the impact of altering individual parameters on model results. In line with the 2020 version of the CGPE, the drug price range was determined by the highest and lowest prices offered by various drug manufacturers on the Hunan Public Resources Trading Service Platform. Due to centralized drug procurement in public hospitals in China, the price of generic drugs was much lower than the corresponding original drugs, resulting in wide price ranges for certain drugs. Other parameter ranges were either obtained from published sources or estimated to be within ±20% of the base-case value. Monte Carlo simulation was used for probabilistic sensitivity analysis, with 1,000 iterations, drawing randomly from pre-specified distributions. As outlined in Table 2, we used gamma distributions for costs, body surface area and body weight, and beta distributions for utility parameters and probabilities.

### 2.5 Scenario analysis

A scenario analysis was conducted to assess the reliability of the model when change the time frame. Consequently, a 20-year time horizon was set, with the death rate for patients in this model being over 99%.

## 3 Results

### 3.1 Base-case and subgroup analysis

Patients who were treated with SINT + Chemo achieved 1.12 QALYs at a cost of $27503.91, while those who received chemotherapy alone achieved 0.8 QALYs at a cost of $19515.48 (Table 3). As a result, the ICER was calculated to be $25239.29/QALY, lower than the WTP threshold of $38223.34. Therefore, SINT + Chemo was considered as the more cost-effective option for treating GC/GCJC as a first-line treatment.

**TABLE 3:** Results of the base-case, subgroup, and scenario analyses.

Group	Total cost ($)	Incremental costs ($)	Overall QALYs	Incermental QALYs	ICER ($/QALYs)
Base-case analysis					
SINT + Chemo group	27503.91	7988.43	1.12	0.32	25239.29
Chemo group	19515.48		0.8		
Subgroup analysis					
PD-L1 CPS≥5					
SINT + Chemo group	34940.60	12980.27	1.36	0.49	26341.01
Chemo group	21960.33		0.87		
PD-L1 CPS<5					
SINT + Chemo group	20518.20	3391.00	0.87	0.19	17658.26
Chemo group	17127.20		0.68		
Scenario analysis					
SINT + Chemo group	28956.58	8747.11	1.19	0.36	24544.15
Chemo group	20209.47		0.83		

Chemo, chemotherapy; SINT, sintiliamb; CPS, combined positve score; ICER, increment cost-effectiveness ratio; QALY, quality-adjusted life-years.

Subgroup analysis results are also shown in Table 3. The incremental costs for SINT + Chemo group with PD-L1 CPS ≥5 and CPS <5 were $12980.27 and $3391, respectively. The incremental effects were 0.49 QALYs and 0.19 QALYs, leading to ICERs of $26341.01/QALY and $17658.26/QALY, respectively, both below the WTP threshold.

### 3.2 Sensitivity analysis

**FIGURE 2:**
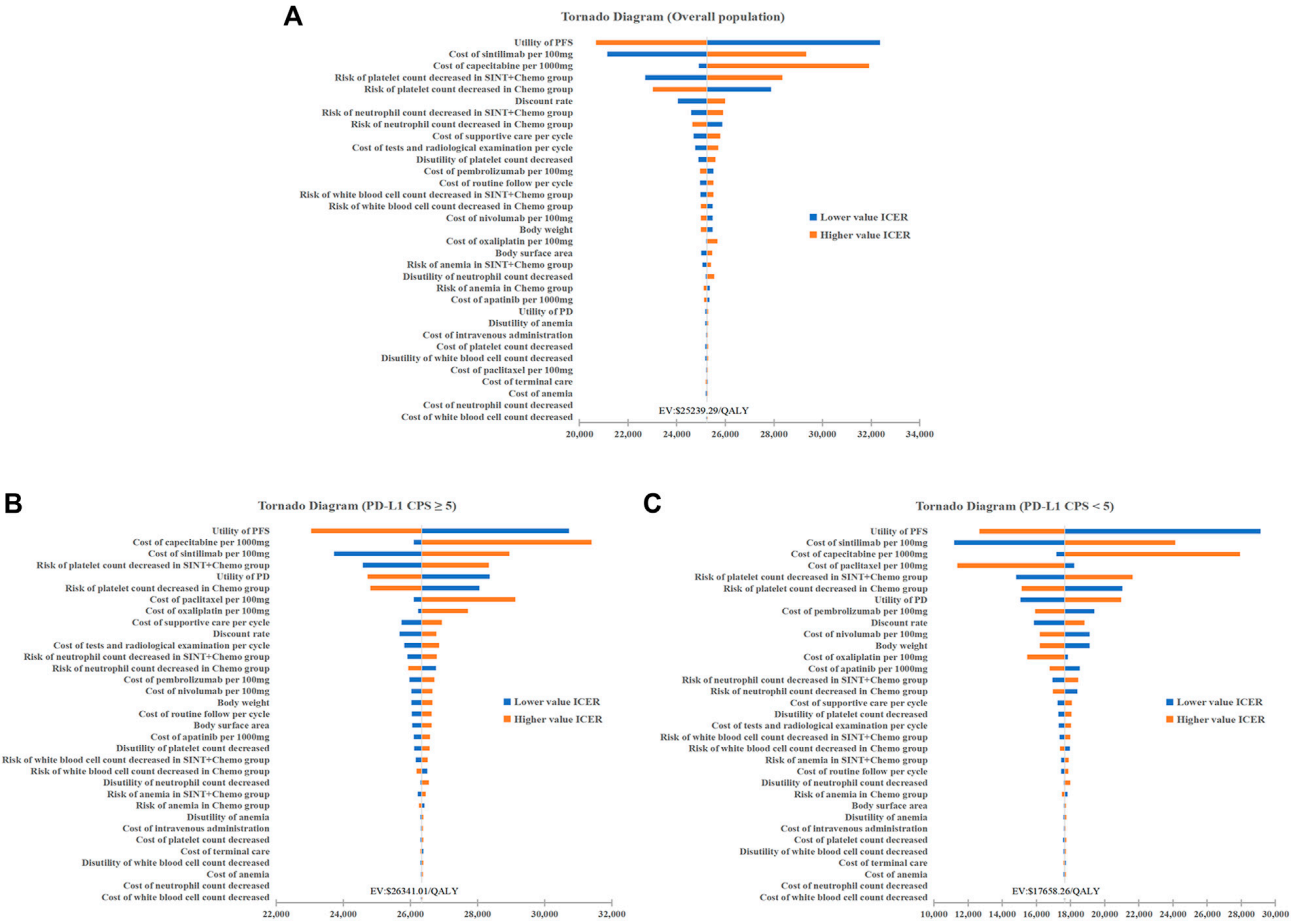
One-way Sensitivity Analysis. **(A)** Overall population. **(B)** Patients with PD-L1 CPS≥5. **(C)** Patients with PD-L1 CPS<5. Chemo, chemotherapy; SINT, sintiliamb.

The tornado diagrams exhibit the results of the one-way sensitivity analysis (Figure 2). It was evident that, in the base-case analysis, the utility value of PFS impacted results the most, with the price of sintilimab and capecitabine following closely behind. The subgroup analysis yielded comparable results to the base-case analysis. The findings from the model were found to be robust, as all ICERs staying below the cost-effective WTP threshold when parameters were adjusted within a defined range.

**FIGURE 3:**
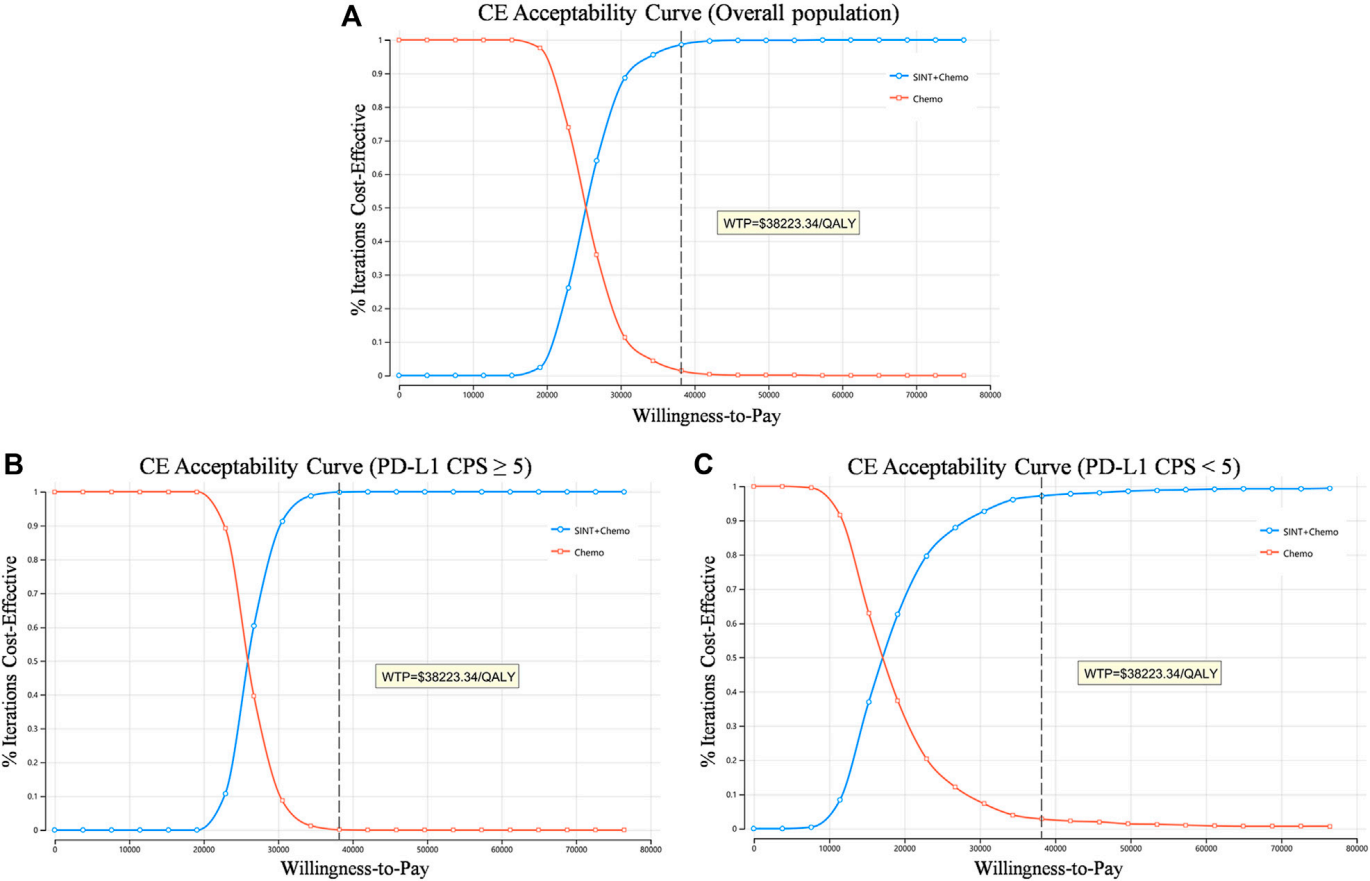
Cost-effectiveness acceptability curve. **(A)** Overall population. **(B)** Patients with PD-L1 CPS≥5. **(C)** Patients with PD-L1 CPS<5. QALY, quality-adjusted life-year; CE, cost-effectiveness; WTP, willingness to pay.

Based on the cost-effectiveness acceptability curves resulting from the probabilistic sensitivity analysis, the likelihood that SINT + Chemo was more cost-effective rose with higher WTP thresholds (Figure 3). When the WTP threshold was $38223.34, the likelihood of SINT + Chemo being cost-effective vs. chemotherapy was 98.6% for the overall population, 99.9% for patients with PD-L1 CPS ≥5, and 97.2% for patients with PD-L1 CPS <5 (Supplementary Figures S4–S6).

### 3.3 Scenario analysis

Over a 20-year time frame, 1.19 QALYs were gained at a cost of $28956.58 in SINT + Chemo group, whereas 0.83 QALYs were gained at a cost of $20209.47 in chemotherapy group. The corresponding ICER was $24544.15/QALY. The conclusion remained in line with the base-case analysis (Table 3).

## 4 Discussion

This research indicates that SINT + Chemo (vs. chemotherapy) was cost-effective as a first-line therapy for advanced GC/GEJC. The ICER was $25239.29/QALY, significantly lower than the Chinese WTP threshold of $38223.34. Since evidence has established that the increased levels of PD-L1 expression correlate with the improved therapeutic effects in ICI treatment (Burtness et al., 2019; Shitara et al., 2020; Janjigian et al., 2021), which is also the case for the ORIENT-16 trial, subgroup analysis has been conducted in the current study. Reassuringly, this study revealed consistent conclusions across two subgroups (PD-L1 CPS ≥5 and CPS <5) that SINT + Chemo was more cost-effective. Despite the two cohorts with PD-L1 CPS <5 not having a significant difference in OS, SINT + Chemo regimen was still more preferable from an economy perspective. This may be largely attributed to the significantly prolonged PFS in the SINT + Chemo group that yielded more QALYs.

Recent economic evaluations of nivolumab or pembrolizumab as the first-line treatment for advanced stomach cancer indicate that neither drug is cost-effective in China. Lang Y et al. performed an evaluation model that showed pembrolizumab in combination with chemotherapy (vs. chemotherapy) was not a cost-effective option for the treatment of advanced gastric cancer both in the US and in China. However, in the US, pembrolizumab alone was deemed cost-effective for patients with PD-L1 CPS ≥10 (Lang et al., 2023). Other published studies, based on the CheckMate-649 trial, examined the economy of nivolumab for advanced GC/GEJC and esophageal adenocarcinoma. According to Morimoto K et al.'s study, the ICER of nivolumab plus chemotherapy vs. chemotherapy exceeded the Japanese WTP threshold (Morimoto et al., 2023). A similar study conducted by Cao X et al. indicated that in the US, nivolumab plus chemotherapy was not preferable to chemotherapy in overall population, as well as in patients with PD-L1 CPS ≥5 and CPS ≥1 (Cao et al., 2023). Other investigations focusing on nivolumab in China led to similar conclusions (Jiang et al., 2022; Shu et al., 2022; Zhang et al., 2023). The latest study conducted by Li W et al. showed that in patients with PD-L1 positive (tumor area positivity score≥ 5%), the combination of tislelizumab and chemotherapy was cost-effective in China at a WTP threshold of 3 times GDP *per capita*, but there was a lack of research on PD-L1 negative patients (Li W et al., 2024). One-way sensitivity analyses in these studies revealed that the most impactful variables influencing the outcomes were the utility of PFS, the utility of PD and the price of ICI, consistent with our findings. This confirms the accuracy of our model to some extent. Hence, the results of our study indicate that, sintilimab stands out as the sole ICI demonstrated to be cost-effective for GC/GEJC regardless of the PD-L1 expression levels thus far, attributed to its favorable clinical outcomes and comparatively modest pricing.

One strength of the model is that the patients enrolled in the ORIENT-16 trial were from 62 centers, all in China. This helps to reduce bias stemming from the geographical or genetic heterogeneity of GC/GEJC (Li Y et al., 2024). Moreover, employing the PartSA model is advantageous as it reduces reliance on assumptions by directly obtaining the patients’ proportion in different health states from OS and PFS curves. Specifically, the proportion of patients in the PFS state was derived from the area under the PFS curve, while the proportion in the PD state was determined by the disparity between the OS and PFS curves. This approach relies on the available survival curves and yielded results that closely align with the actual observed data, making it a popular choice for assessing the cost-effectiveness of anti-tumor medications in comparison to Markov model.

This study also has several limitations. Firstly, the economics of other similar ICIs that have shown favorable clinic benefits are not compared in this study, such as nivolumab, pembrolizumab, sugemalimab, and tislelizumab. Considering the absence of direct comparative clinical data, a potential economic evaluation through meta-analysis can be conducted in the future when sufficient data becomes available. Secondly, the study’s accuracy may be affected by the uncertainty resulting from extrapolating survival curves. Nonetheless, there is currently no established methodology to resolve this issue perfectly. Real-world and long-term follow-up data are needed to verify the outcomes. Thirdly, it is important to mention that the utility values utilized in this research are derived from previous studies and may differ from the ORIENT-16 trail. Fourthly, the cost and utility calculations did not take into account grade 1–2 adverse events and long-term side effects, which may have resulted in an underestimate of the costs and disutility values. Lastly, the estimated ranges of serious adverse event risks in this study were set to be within ±20% of the base-case value. However, this may not accurately reflect the uncertainty associated with the parameters. Nonetheless, the results of the one-way sensitivity analysis indicated a relatively minor impact of these parameters on the outcome.

In conclusion, irrespective of PD-L1 expression levels, SINT + Chemo is more cost-effective than chemotherapy alone as a first-line treatment for unresectable advanced or metastatic GC/GEJC in China. These findings will help physicians to establish appropriate treatment protocols for their patients and hold significant implications for healthcare decision-making.

## Data Availability

The original contributions presented in the study are included in the article/Supplementary Material, further inquiries can be directed to the corresponding author.
