# Supplementary material for: Cost-effectiveness analysis of first-line sintilimab plus chemotherapy vs. chemotherapy alone for unresectable advanced or metastatic gastric or gastroesophageal junction cancer in China
Source: Front Pharmacol. 2024 Sep 4;15:1411571. doi: 10.3389/fphar.2024.1411571 (PMC11408219; doi:10.3389/fphar.2024.1411571)
Supplement: Supplementary file 1 [file DataSheet1.PDF]

Supplementary Material

1 Supplementary Tables

Supplementary Table 1. CHEERS Checklist (2022)

Topic	No.	Item	Reported?
**Title and abstract**			
Title	1	Identify the study as an economic evaluation and specify the interventions being compared.	Yes
Abstract	2	Provide a structured summary that highlights context, key methods, results, and alternative analyses.	Yes
**Introduction**			
Background and objectives	3	Give the context for the study, the study question, and its practical relevance for decision making in policy or practice.	Yes
**Methods**			
Health economic analysis plan	4	Indicate whether a health economic analysis plan was developed and where available.	Yes
Study population	5	Describe characteristics of the study population (such as age range, demographics, socioeconomic, or clinical characteristics).	Yes
Setting and location	6	Provide relevant contextual information that may influence findings.	Yes
Comparators	7	Describe the interventions or strategies being compared and why chosen.	Yes
Perspective	8	State the perspective(s) adopted by the study and why chosen.	Yes
Time horizon	9	State the time horizon for the study and why appropriate.	Yes
Discount rate	10	Report the discount rate(s) and reason chosen.	Yes
Selection of outcomes	11	Describe what outcomes were used as the measure(s) of benefit(s) and harm(s).	Yes
Measurement of outcomes	12	Describe how outcomes used to capture benefit(s) and harm(s) were measured.	Yes
Valuation of outcomes	13	Describe the population and methods used to measure and value outcomes.	Yes
Measurement and valuation of resources and costs	14	Describe how costs were valued.	Yes
Currency, price date, and conversion	15	Report the dates of the estimated resource quantities and unit costs, plus the currency and year of conversion.	Yes
Rationale and description of model	16	If modelling is used, describe in detail and why used. Report if the model is publicly available and where it can be accessed.	Yes

Topic	No.	Item	Reported?
Analytics and assumptions	17	Describe any methods for analysing or statistically transforming data, any extrapolation methods, and approaches for validating any model used.	Yes
Characterising heterogeneity	18	Describe any methods used for estimating how the results of the study vary for subgroups.	Yes
Characterising distributional effects	19	Describe how impacts are distributed across different individuals or adjustments made to reflect priority populations.	Yes
Characterising uncertainty	20	Describe methods to characterise any sources of uncertainty in the analysis.	Yes
Approach to engagement with patients and others affected by the study	21	Describe any approaches to engage patients or service recipients, the general public, communities, or stakeholders (such as clinicians or payers) in the design of the study.	Not applicable
**Results**			
Study parameters	22	Report all analytic inputs (such as values, ranges, references) including uncertainty or distributional assumptions.	Yes
Summary of main results	23	Report the mean values for the main categories of costs and outcomes of interest and summarise them in the most appropriate overall measure.	Yes
Effect of uncertainty	24	Describe how uncertainty about analytic judgments, inputs, or projections affect findings. Report the effect of choice of discount rate and time horizon, if applicable.	Yes
Effect of engagement with patients and others affected by the study	25	Report on any difference patient/service recipient, general public, community, or stakeholder involvement made to the approach or findings of the study	Not applicable
**Discussion**			
Study findings, limitations, generalisability, and current knowledge	26	Report key findings, limitations, ethical or equity considerations not captured, and how these could affect patients, policy, or practice.	Yes
Other relevant information			
Source of funding	27	Describe how the study was funded and any role of the funder in the identification, design, conduct, and reporting of the analysis	Yes
Conflicts of interest	28	Report authors conflicts of interest according to journal or International Committee of Medical Journal Editors requirements.	Yes

From: Husereau, D., Drummond, M., Augustovski, F., de Bekker-Grob, E., Briggs, A. H., Carswell, C., et al. (2022). Consolidated health economic evaluation reporting standards 2022 (CHEERS 2022) statement: Updated reporting guidance for health economic evaluations. *MDM Policy Pract.* 7(1)**,** 23814683211061097. doi:10.1177/23814683211061097

Supplementary Table 2. Summary of the statistical goodness-of-fit of Kaplan Meier survival curves.

	Exponential	Weibull	Gamma	Generalized gamma	Gompertz	Log-normal	Log-logistic
Overall population							
SINT+ Chemo OS curve							
AIC	1463.441	1449.955	1446.745	1444.652	1461.534	1444.353	1440.581
BIC	1467.231	1457.535	1454.325	1456.022	1469.114	1451.933	1448.161
Chemo OS curve							
AIC	1598.066	1571.747	1567.038	1564.527	1588.77	1563.947	1562.484
BIC	1601.844	1579.302	1574.594	1575.860	1596.326	1571.503	1570.040
SINT+ Chemo PFS curve							
AIC	1346.805	1342.176	1338.17	1332.187	1348.735	1336.559	1321.179
BIC	1350.595	1349.756	1345.75	1343.557	1356.315	1344.139	1328.758
Chemo PFS curve							
AIC	1557.261	1529.815	1518.857	1509.459	1555.162	1510.925	1501.389
BIC	1561.039	1537.370	1526.413	1520.792	1562.718	1518.480	1508.945
Patients with PD-L1 CPS≥5							
SINT+ Chemo OS curve							
AIC	800.371	794.914	792.785	789.746	801.397	787.748	788.728
BIC	803.654	801.480	799.352	799.596	807.963	794.314	795.294
Chemo OS curve							
AIC	981.680	972.485	969.905	967.496	980.523	965.697	966.241
BIC	984.979	979.082	976.502	977.391	987.120	972.294	972.838
SINT+ Chemo PFS curve							
AIC	821.738	816.839	812.548	801.604	823.734	799.907	799.815
BIC	825.021	823.406	819.115	811.454	830.301	806.474	806.381
Chemo PFS curve							
AIC	939.638	925.171	916.985	903.885	940.510	902.137	901.006
BIC	942.937	931.768	923.582	913.780	947.107	908.734	907.603
Patients with PD-L1 CPS<5							
SINT+ Chemo OS curve							
AIC	653.482	643.810	643.083	644.992	648.610	646.944	642.979
BIC	656.349	649.545	648.818	653.595	654.345	652.679	648.714
Chemo OS curve							
AIC	618.351	597.408	595.455	596.813	606.519	595.848	595.355
BIC	621.164	603.032	601.079	605.249	612.144	601.472	600.979
SINT+ Chemo PFS curve							
AIC	526.847	527.281	526.139	525.223	528.674	526.194	519.962
BIC	529.715	533.017	531.874	533.826	534.409	531.929	525.697
Chemo PFS curve							
AIC	615.437	601.256	597.538	596.028	611.997	594.304	595.879
BIC	618.249	606.881	603.163	604.465	617.622	599.929	601.504

Chemo, chemotherapy; SINT, sintiliamb; OS, overall survival; PFS, progression-free survival; AIC, Akaike’s information criterion; BIC, Bayesian information criterion.

Supplementary Table 3. Subsequent treatment after disease progression

Treatment	rate		usage and dosage
	SINT+Chemo	Chemo	
capecitabine	24.42%	22.70%	1000 mg/m^2 orally twice a day for days 1-14 every 3weeks
oxaliplatin	10.29%	10.20%	130 mg/m^2 was administered intravenously every 3 weeks
paclitaxel	40.86%	40.35%	80mg/m^2 was administered intravenously for days 1,8,15 every 28 days
apatinib	14.13%	15.24%	850mg orally once a day
pembrolizumab	5.15%	5.76%	200mg was administered intravenously every 3 weeks
nivolumab	5.15%	5.76%	3mg/kg was administered intravenously every 14 days

Chemo, chemotherapy; SINT, sintiliamb.

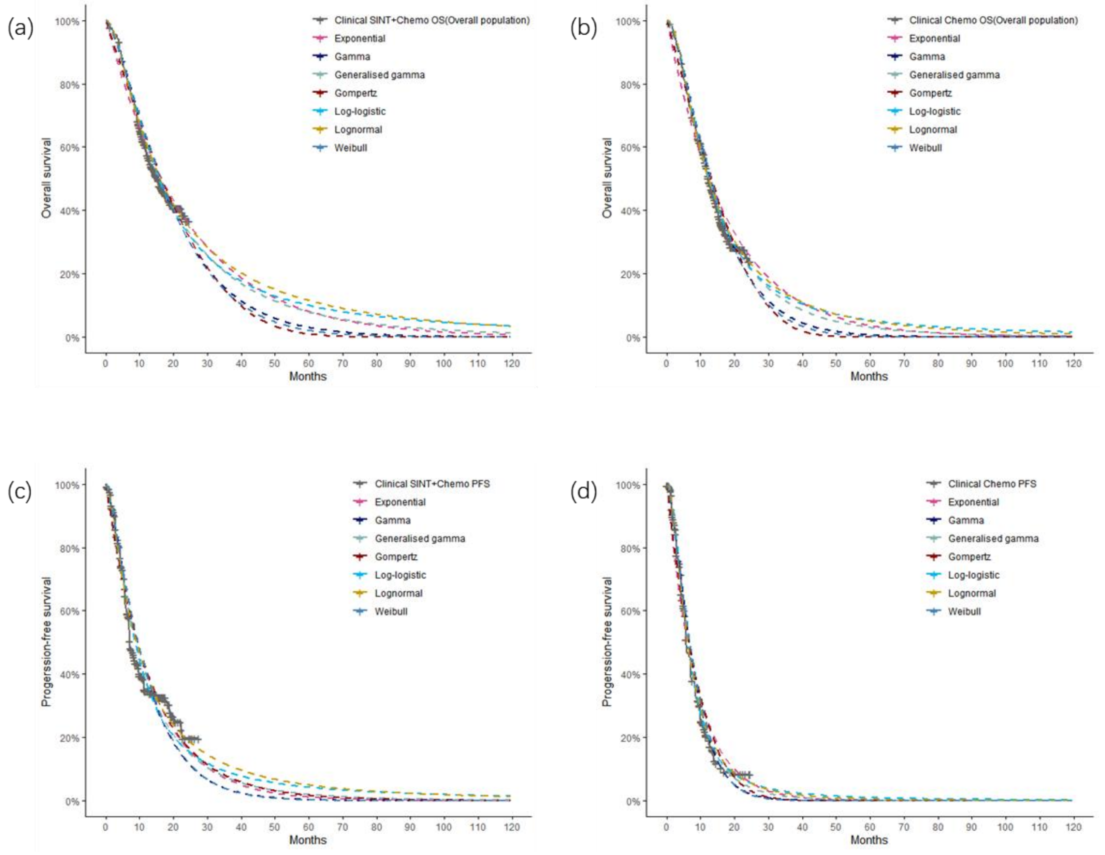

Supplementary Figure 1. Fitting and extrapolation of Kaplan Meier survival curve for overall patients. (a) The results of OS curve in SINT+ Chemo arm. (b) The results of OS curve in Chemo arm. (c) The results of PFS curve in SINT+ Chemo arm. (b) The results of PFS curve in Chemo arm. Chemo, chemotherapy; SINT, sintiliamb; OS, overall survival; PFS, progression-free survival.

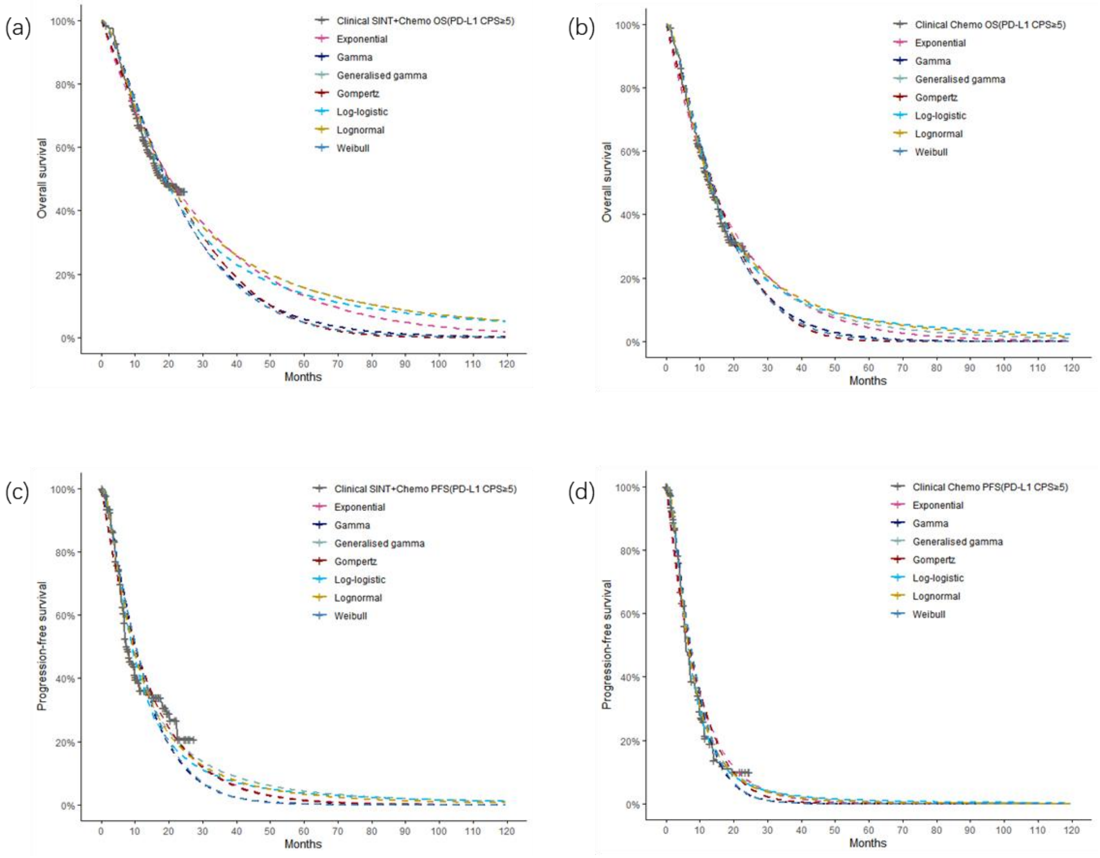

Supplementary Figure 2. Fitting and extrapolation of Kaplan Meier survival curve for patients with PD-L1 CPS≥5. (a) The results of OS curve in SINT+ Chemo arm. (b) The results of OS curve in Chemo arm. (c) The results of PFS curve in SINT+ Chemo arm. (b) The results of PFS curve in Chemo arm. Chemo, chemotherapy; SINT, sintiliamb; OS, overall survival; PFS, progression-free survival.

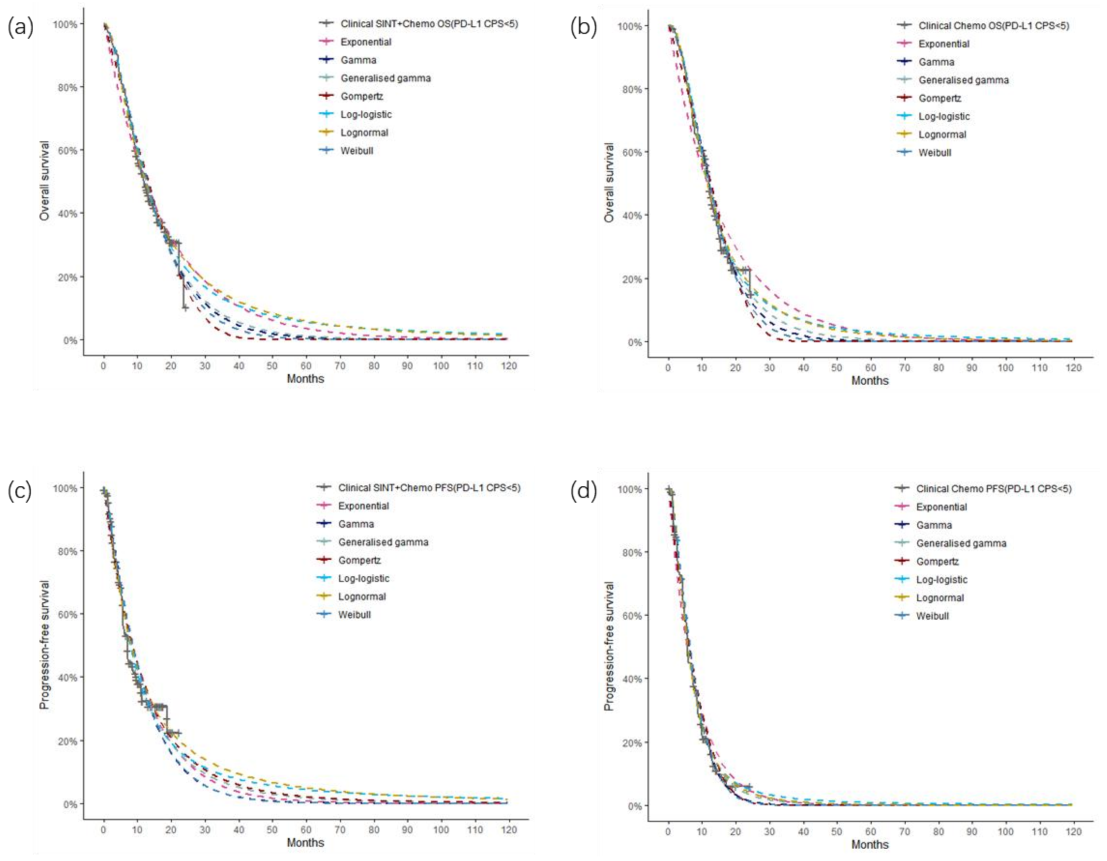

Supplementary Figure 3. Fitting and extrapolation of Kaplan Meier survival curve for patients with PD-L1 CPS<5. (a) The results of OS curve in SINT+ Chemo arm. (b) The results of OS curve in Chemo arm. (c) The results of PFS curve in SINT+ Chemo arm. (b) The results of PFS curve in Chemo arm. Chemo, chemotherapy; SINT, sintiliamb; OS, overall survival; PFS, progression-free survival.

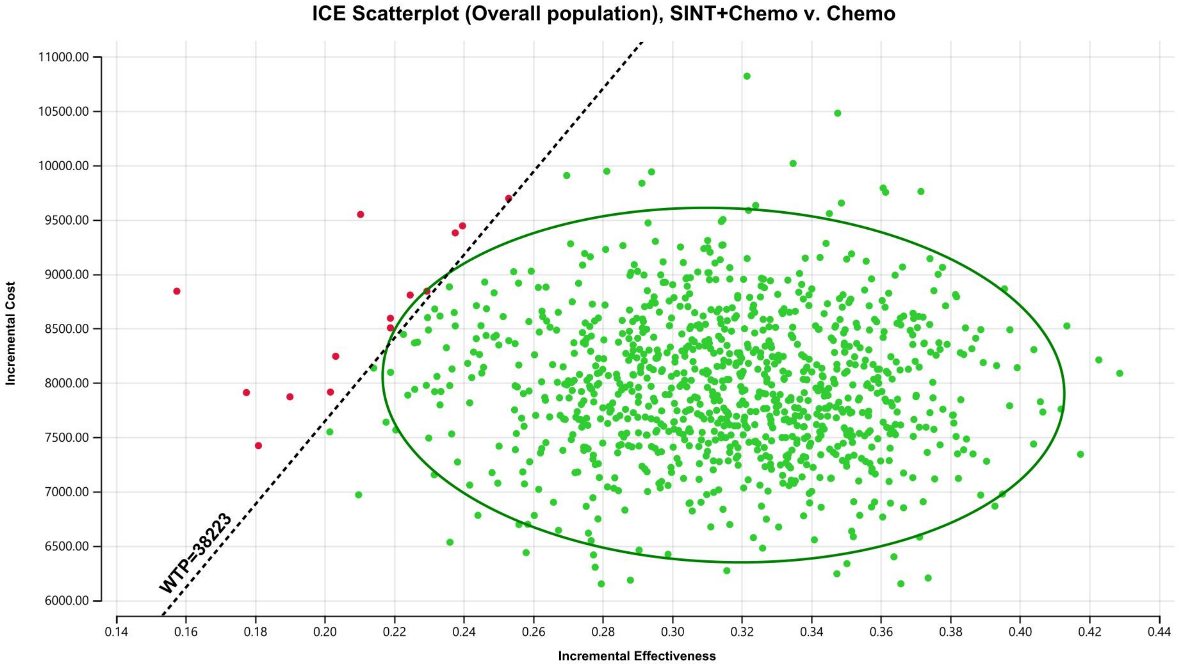

Supplementary Figure 4. Scatter plot in the probabilistic analysis for overall patients. ICE, incremental cost-effectiveness; Chemo, chemotherapy; SINT, sintiliamb.

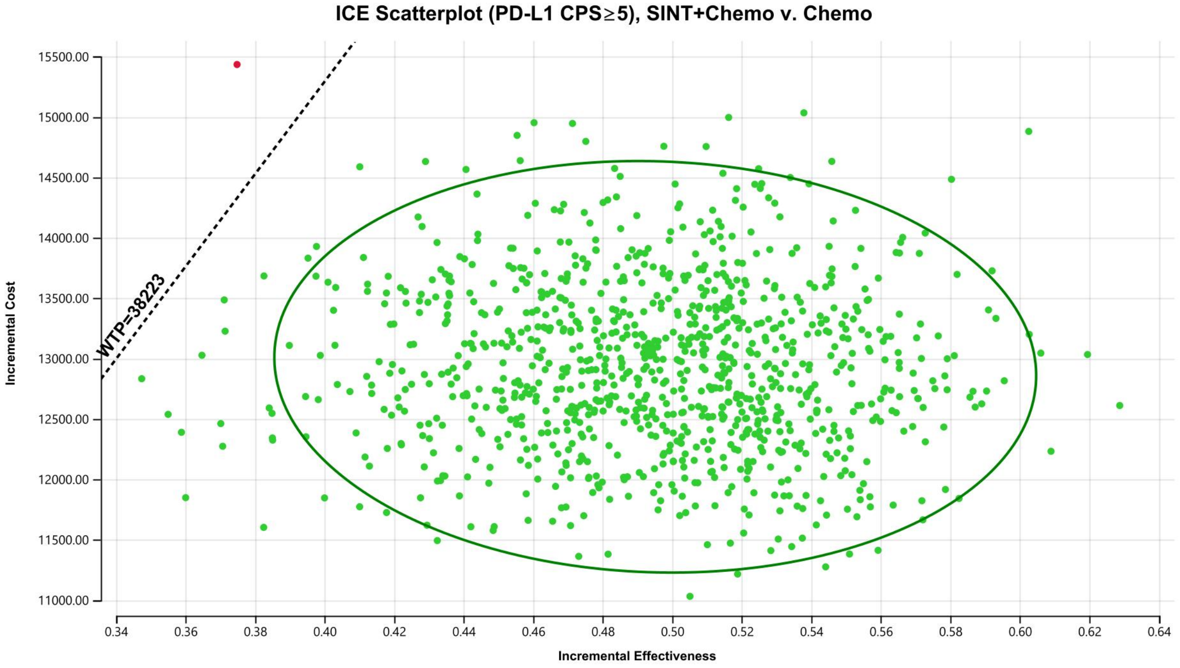

Supplementary Figure 5. Scatter plot in the probabilistic analysis for patients with PD-L1 CPS≥5. ICE, incremental cost-effectiveness; Chemo, chemotherapy; SINT, sintiliamb.

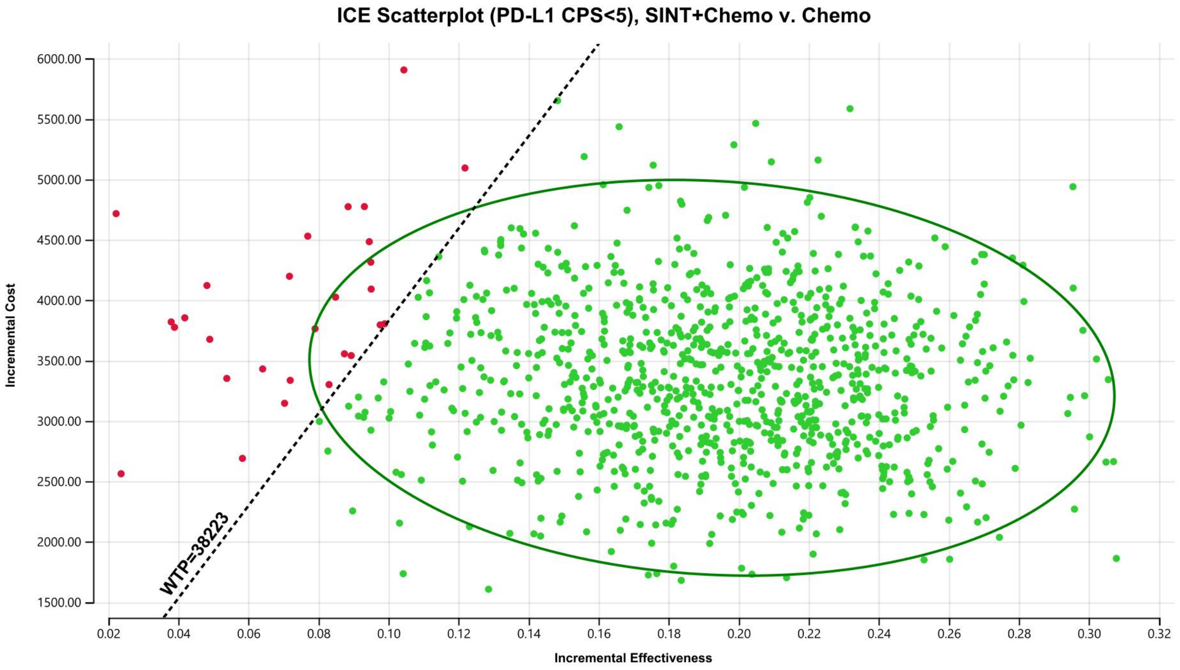

Supplementary Figure 6. Scatter plot in the probabilistic analysis for patients with PD-L1 CPS<5. ICE, incremental cost-effectiveness; Chemo, chemotherapy; SINT, sintiliamb.
